# Obstetrics

**Published:** 1906-06

**Authors:** Walter C. Swayne


### OBSTETRICS.

The occurrence of **suppuration in the female pelvis** is sufficiently common to place the desirability of a critical examination of views as to its causation, pathological anatomy and treatment beyond question.

Grad[2] describes pelvic suppurations as falling into one of two broad anatomical divisions according as they are intra- or extra-peritoneal. Both of these conditions may be present at the same time, and in quite a large proportion of cases it is difficult in the extreme to distinguish between them clinically. For the production of each variety different avenues of infection are to some extent responsible, and this renders the consideration of the various points as to diagnosis, prognosis and treatment liable to considerable variation. There is now no question as to the relative frequency of the two varieties, and, contrary to the teaching of a few decades ago, the intra-peritoneal form is generally recognised as being infinitely the more common. The avenues of infection in the intra-peritoneal variety are mainly two, namely that from without by way of the genital tract, and that in which the infection gains the pelvic cavity from some viscus outside the generative organs, or through some other route than that of the vagina, uterus or Fallopian tubes.

[1] *Lancet*, 1906, i. 82. [2] Grad, H., *Am. J. Obst.*, 1906, liii. 239.

The first route is responsible for all those cases in which pus forms as the result of septic or specific (*i.e.* gonorrhœal) infection, but in those cases in which suppuration occurs in cysts of the ovary or broad ligament, fibroids, hæmotocele, tubal mole, in blood collections, the result of menstrual refluxes, or as the result of appendicitis, intestinal perforations or growths, or as the result of tubercular diseases, the avenue of infection is a different one.

Infections of the genital tract with specific micro-organisms lead by continuity of tissue to a progressive infection of the endometrium, Fallopian tubes, ovaries and peritoneum adjacent to the fimbriated extremities of the tubes, so that in gonorrhœal infections, especially if unaccompanied by pyogenic organisms, the inflammatory reaction will be confined to the endometrium, Fallopian tube and ovary, and the peritoneum immediately surrounding these structures. Pus collections in these cases are not so frequent, in spite of the marked local affection of the peritoneum produced, as in infections caused by the pyogenic micro-organisms, but remote effects, such as arthritis and endocarditis, are common, and, if a mixed infection occurs an intra-peritoneal suppuration is fairly certain. In pyogenic infections the route of infection is by the lymphatics as well as by tissue continuity, and as a rule these result from puerperal infection, as the result of abortion, especially criminal abortion, the use of dirty instruments, or intra-uterine applications with faulty aseptic technique.

In pyogenic infections, again, the avenues being double, much wider areas are affected, more rapid invasion occurs, and the symptoms are more acute. Adhesions form over wider areas, and involve not only the ovaries and tubes, but omentum and coils of intestine. Between these adhesions pus collections form, giving rise to abscesses of various dimensions.

Therapeutic measures designed for the relief of suppuration due to pyogenic infections necessarily involve the evacuation of each and all of the pus collections formed. So that while gonorrhœal infection may produce acute peritonitis, it is more usual for the effects to be localised, while in the pyogenic cases more diffused suppuration and multiple abscesses are the usual result. The pyogenic infections are most usually due to the streptococcus, but the colon bacillus is not infrequently found. The staphylococcus aureus and albus are only found uncommonly. In all these cases when the acute symptoms have subsided the pus after a time is found to be sterile.

Another method of causation of intra-peritoneal suppuration is from punctures of the uterus which is especially apt to be found in criminal abortion. A noteworthy point is that in a large number of these cases the wound in the uterus will have healed and no trace of the actual source of infection remain.

Extra-peritoneal collections of pus are due either to infection by way of lymphatics from wounds of the perineum, vagina and uterus, or to hæmatomata of the broad ligaments, caused either by rupture of ectopic pregnancies or of enlarged veins in the broad ligaments. The tendency for these collections is to track outwards and appear on the surface of the abdomen immediately above Poupart's ligament or occasionally at the sciatic notch. They may even point in Scarpa's triangle.

The general conclusions as to treatment seem to be as follows: (1) That opening from the vagina is to be preferred at any rate in acute cases. (2) That if the abdominal route is selected, operation during the acute stages is to be avoided if possible since sterilisation of the pus will eventually occur and reduce the risk of operation to a minimum. (3) That in any case all pus collections must be evacuated. This is often difficult, and even impossible, if the vaginal route alone is made use of.

Adam[1] remarks that whether an inflammation will pass through its various phases to that of suppuration depends on the dosage and virulence of the infection and the resistance of the patient. He states that while inflammation of the tubes is the most common cause of pelvic suppuration, the other situations in which suppuration may be found are numerous. He states that in a large number of cases in his experience the ampulla has been found dilated with pus while the isthmus of the tube was healthy. In several of these cases, moreover, the endometrium showed traces of infection, so that while both extremities of the tract were affected the middle portion remained apparently free, although it must have been traversed by the infection. Probably this is due to the differences of blood and lymphatic supply in the two portions of the tube. Adam takes the view that the vast majority of cases of pelvic suppuration are due to gonorrhœa, this view being dissimilar to that of Grad, who regards gonorrhœa as the less common cause of suppurations.

A large number of cases, however, arise as the result of septic infection after parturition at full time or abortion. Tuberculosis as a cause is responsible for a certain though comparatively small number of cases. Experience shows that it is not as a rule the severer cases of septic puerperal infection that produce pelvic suppuration, but those cases in which the infection is of a comparatively mild degree. For example, a mild septic infection produces comparatively slight pelvic inflammation, followed by adhesions and closure of the inflamed tube, whilst an attack of influenza, or some other variety of general fever results in a pelvic suppuration. It may be months after the first attack, from infection of the damaged area with the colon bacillus through the medium of theintestinal adhe-sions.

This is particularly apt to occur as the result of an attack of appendicitis in a case in which a right-sided inflammation has

[1] Adam, *J. Obst. and Gynæc. Brit. Emp.*, 1906, ix. 35.

led to adhesions between the appendix and the tube. The appendix has been actually found to open into an occluded tube into which it was discharging pus.

The phenomena attending abortion are well known to predispose to septic infection in a very high degree, and unless the treatment of this condition is carried out with the greatest care to maintain asepsis in cases in which manual or instrumental interference is necessary, infection, generally of a mild type, is a very common result, with damage to the appendages and, in a certain number of cases, suppuration as an immediate or remote sequela.

As a result of the foregoing Adam insists on the importance of examining parturient patients not only during the puerperal period, but after the normal period of involution has passed, and more especially all those patients in whom the puerperal period has shown any irregularity. In abortion the complete emptying of the uterus as early as possible is of the greatest importance. No woman with pelvic adhesions attaching the tubes to intestine is ever safe from suppuration. The lines of defence lie in greater care as to aseptic technique during parturition, and a closer observation of the puerperal period, as the first, and post-puerperal examination, at any rate in every case in which there has been any irregularity in pulse-rate, temperature, or condition of the lochia during the puerperium as the second.

Worrall[1] in dealing with the treatment of pelvic suppuration bases his conclusions on his own experience, and remarks that out of 111 cœliotomies performed by himself forty-five were necessitated for the relief of suppuration in the pelvis. Of twenty-four bacterial examinations, in nine the pus was sterile, streptococcus was found in five, bacillus coli in five, gonococcus in four, and strepto- and pneumococcus in one. The origin of the trouble appeared to be gonorrhœal in fourteen, parturition and abortion fourteen, criminal abortion and trauma five, suppurating ovarian cysts two, tubercle two. In only three out of the forty-five was there cellulitis with pus tubes, and in *one* suppurative cellulitis *without* pus tubes. In each case curetting was done at the same sitting as the section.

In ten cases the uterus was removed with the appendages, and in seven the appendix. Suppuration of the laparotomy wound occurred four times. Vaginal cœliotomy was done as a preliminary in forty-four cases, and in four was the only operation.

Worrall advocates the systematic employment of both the vaginal and abdominal routes. He lays down the following lines of treatment: (1) All cases of pelvic suppuration demand operation. (2) If patient is desperately ill operation should be immediate, unless improvement seems after careful examination to be likely to result with rest, &c., when postponement until the acute symptoms have subsided may be advisable.

[1] Worrall, *Brit. Gynæc. J.*, 1905-6, xxi. 258.

(3) Curettage and vaginal cœliotomy precede the abdominal operation in all cases. In cases in which severe constitutional symptoms are absent the abdominal operation is proceeded with at once, in acute cases after an interval. (4) The uterus is removed, as is the appendix, in every case in which these organs are definitely infected. (5) Flushing is avoided except in general purulent peritonitis. (6) Drainage into the vagina is used invariably.

Tuffier[1] pleads for the retention of the uterus in operations for old pelvic suppurations of the tubes; he removes the diseased tubes only, leaving the uterus and ovaries whenever possible, *i.e.* whenever these are comparatively healthy. Curettage of the uterus he considers sufficient, but destroys the uterine end of the resected tube with the actual cautery. He commences his resection of the diseased tube at the uterine cornu and dissects it from the broad ligament outwards.

J. W. Taylor,[2] discussing the value of colpotomy in the thrombotic form of puerperal fever, advocates the opening up of the broad ligaments from the vagina in cases in which repeated rigors with the presence of thickened cords in the broad ligaments in a case of septic puerperal infection indicate septic thrombo-phlebitis. He describes three cases, in each of which he found small abscesses at the uterine end of the thrombus; he evacuated the abscesses, and packed the cavities after clearing them out with moist iodoform gauze. He then incises the posterior fornix and explores the pelvis with the finger, perhaps locating other pus collections; he then inserts a gauze draining pack into the pouch of Douglas. In each of his cases success resulted. His claim that this method of treatment is superior to that of opening the abdomen and ligaturing the veins beyond the thrombus and so cutting off the access of septic thrombi to the general circulation, with the production of all the phenomena of pyæmia and the ultimate death of the patient in many instances, or her recovery after weeks of illness and the repeated opening of metastatic abscesses, would appear to be well founded.

Porache[3] advocates the treatment of cases of suppuration in the pelvis, whether of connective tissue in the peritoneum or pyosalpinx, by posterior colpotomy. He considers that laparotomy or vaginal cœliotomy with removal of the diseased parts are unjustifiable.

Morian[4] advocates the evacuation of pus collections in the pouch of Douglas into the rectum by means of a trocar about $6\frac{3}{4}$ inches in length, which he passes through the anterior wall of

[1] Tuffier, *Surg. Gynæc. and Obst.*, 1905, i. 209; abstract in *Brit. Gynæc. J.*, 1905-6, xxi. Summary, p. 151.

[2] Taylor, *Brit. Gynæc. J.*, 1905-6, xxi. 75.

[3] Porache, *Zentralbl. f. Gynäk.*, 1905, xxix. 158; *Brit. Gynæc. J.*, 1905-6, xxi. Summary, p. 18.

[4] Morian (Essen-Ruhr), *München. med. Wchnschr.*, 1906, liii. 119; abstract in *Brit. Gynæc. J.*, 1905-6, xxi. Summary, p. 206.

the rectum. He considers this preferable to the vaginal opening as it avoids the possibility of infection of the uterus. He avoids both washing out and drainage.

In reviewing the opinions expressed above, it is to be noted that much more use might have been made of the bacteriologist by several of the writers before making dogmatic statements as to the frequency of the different bacterial causes of pelvic suppuration, notably as to the rôle of the gonococcus. The view which is probably nearest to the truth appears to be that while a large number of suppurations follow gonorrhœal infection, these are by no means all due. primarily to the gonococcus (if the pus-producing power of this organism was so great as seems to be supposed, should not the male sufferers, even after allowing for anatomical differences, suffer in a much larger proportion from perineal abscess, &c.?), but result from its power of lessening tissue resistance to the pyogenic organisms proper.

There is now no doubt as to the relative infrequency of extra-peritoneal suppurations such as true pelvic cellulitis. None of the writers mention the value of a differential blood count in cases in which, with an obviously inflammatory mass in the pelvis, suppuration is not definitely ascertained to be present. An absence, of marked leucocytosis and of increase of poly-morpho-nuclears has prevented the writer from proceeding to explore such a mass, with the result that the whole thing cleared up completely.

There can be no question as to the value of colpotomy even in an acute case. The writer having to treat a case of acute and apparently generalised puerperal peritonitis, after curetting the uterus, incised the posterior cul-de-sac and evacuated a quantity of stinking pus with relief to the acuter symptoms, but without curing the patient. Some days later her condition was so improved, although she was still febrile, as to permit of a laparotomy, when from a mass of adherent intestine several pus collections were evacuated, with the result that all her symptoms subsided and recovery took place. In a similar case posterior colpotomy alone sufficed to cure an acute septic peritonitis after a criminal abortion.

Delay in dealing with these cases is often justifiable and advantageous, but the acuter cases can always be attacked from the vagina and drained as a temporary measure; but in this procedure the operator must remember that hemorrhage from the laceration of a vessel may necessitate opening the abdomen. This actually occurred to the writer in dealing with a suppurating parovarian cyst, from a wound of a vessel in its capsule. This patient uneventfully recovered.

WALTER C. SWAYNE.

